# Metastasized pancreatic neuroendocrine tumor in a teenage girl: a case report

**DOI:** 10.1186/s13256-015-0708-3

**Published:** 2015-10-05

**Authors:** Tina Tremmel, Stefan Holland-Cunz, Patrick Günther

**Affiliations:** 1 Department of Pediatric Surgery, University Hospital Heidelberg, INF 110, 69120 Heidelberg, Germany; 2 Department of Pediatric Surgery, University Hospital Basel, Spitalstrasse 33, 4056 Basel, Switzerland

**Keywords:** Functioning pancreatic neuroendocrine tumor (F-PanNET), Islet cell tumors, Non-functioning pancreatic neuroendocrine tumor (NF-PanNET), Pancreatic neuroendocrine tumor (PanNET), Yttrium-90 DOTATOC therapy

## Abstract

**Introduction:**

Metastasized pancreatic neuroendocrine tumors are extremely rare malignancies, especially in children. Therefore, therapeutic options are limited, and few standardized therapy regimens exist.

**Case presentation:**

We report a case of a 14-year-old white girl. In 2011 she was diagnosed with a metastasized, well-differentiated pancreatic neuroendocrine tumor with expression of synaptophysin and chromogranin A. We describe her clinical course with special attention to her individual therapeutic regimens while bringing together several disciplines of medicine.

**Conclusions:**

In patients such as ours, surgical intervention may be the only therapy that will lead to long-term survival.

## Introduction

Metastasized pancreatic neuroendocrine tumors (PanNETs) are rare malignancies, especially in children and young adolescents, for which there is no standard therapeutic regimen. These lesions carry a poor prognosis. In this report, we present a case of a teenage girl (14-years old at age of first presentation) with a metastasized well-differentiated PanNET. A multidisciplinary therapeutic approach was designed by pediatric hematologists-oncologists, pediatric surgeons, visceral surgeons, pediatric radiologists and radio-oncologists. The primary tumor as well as its metastases have been kept in check to date. The patient is in good condition with little limitation of her daily activities.

## Case presentation

In 2010, a 14-year-old white girl presented to our hospital and described having symptoms of ongoing abdominal pain of approximately 3 weeks’ duration that was responsive to analgesic medication. She reported no prior history of nausea, vomiting, diarrhea, fever, sweating or weight loss. Her family history has been uneventful; both parents and a 19-year-old sister are healthy.

**Fig. 1:**
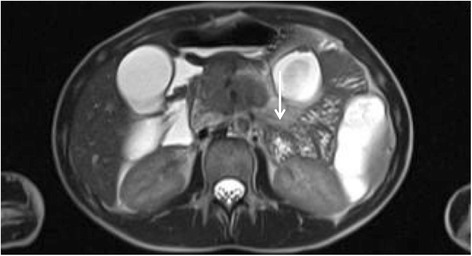
Axial, T2-weighted, half-Fourier single-shot turbo spin-echo hydromagnetic resonance imaging scan of the patient’s abdomen. Retroperitoneal tissue augmentation of the dorsal pancreas is visualized with contrast enhancement (*arrow*)

**Fig. 2:**
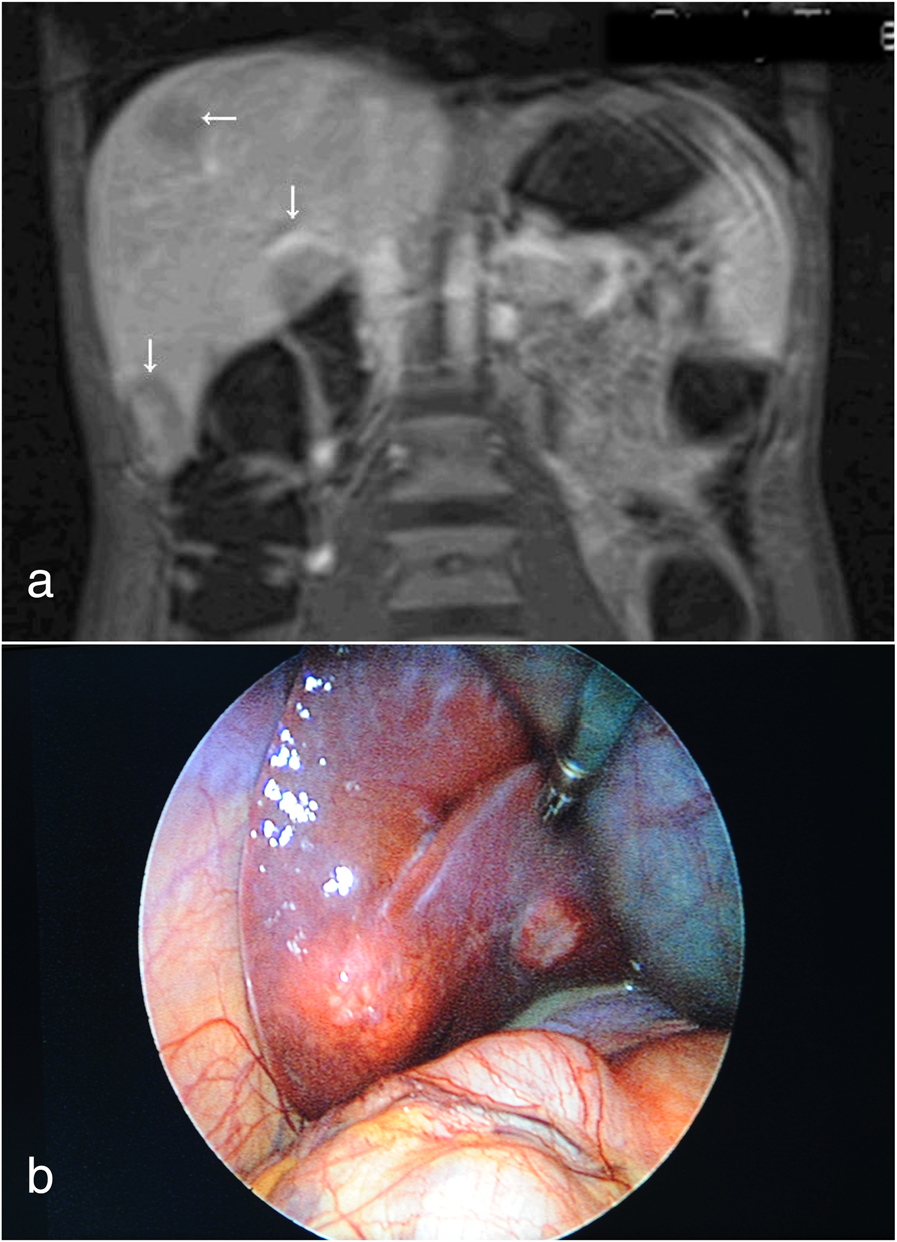
Liver metastases. **a** T1-weighted hydromagnetic resonance imaging scan of the patient‘s abdomen (Gadovist coronary contrast agent; Bayer HealthCare, Berlin, Germany) shows multiple hypodense liver lesions (arrows). **b** Photograph taken during laparoscopy, showing one of the liver metastases

An abdominal ultrasound taken at that time showed normal upper abdominal organs and multiple enlarged lymph nodes that had previously been thought to be a reaction to a nonspecific infection. Her pain increased 2.5 weeks later, resulting in another visit to the emergency room. An abdominal magnetic resonance imaging (MRI) scan revealed a suspicious area in the head of her pancreas, persisting enlarged lymph nodes and unclear liver lesions (Fig. 1 and Fig. 2).

**Fig. 3:**
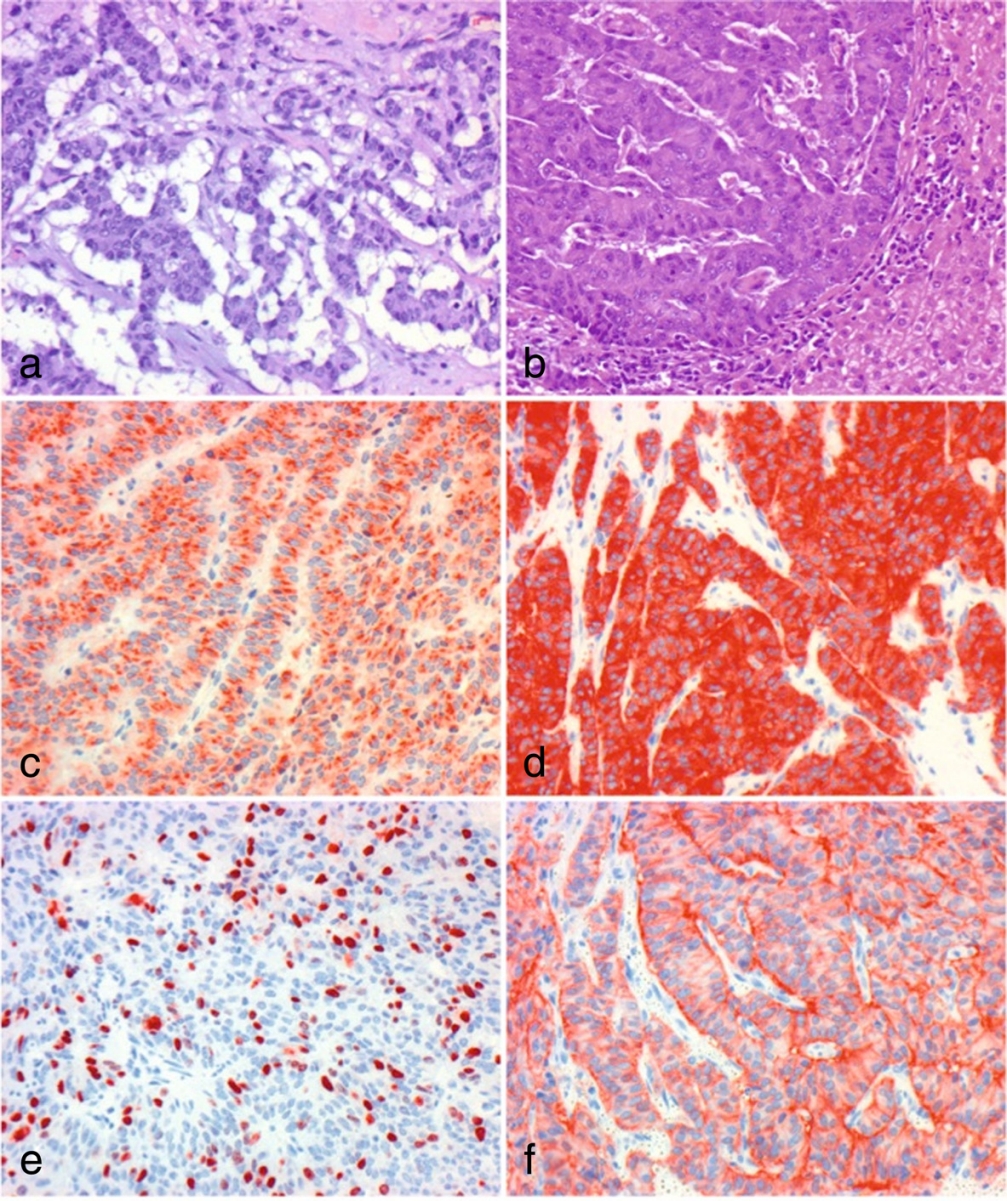
Histopathological findings. Microscopic aspects of primary tumor (**a**) and liver metastasis (**b** through **f**). As shown in hematoxylin and eosin-stained sections (**a**,** b**), the tumor displays an organoid growth pattern with predominant trabecular formations of atypical epithelial cells. Nuclei show only little to moderate pleomorphism, and the cytoplasm is well formed and sometimes contains vacuoles. On immunohistochemical examination, tumor cells show positivity for the neuroendocrine markers chromogranin A (**c**) and synaptophysin (**d**). Ki67 staining (**e**) reveals high proliferative activity. The tumor cells display membranous positivity of the somatostatin receptor 2a (**f**)

A scheduled surgical biopsy was taken after the patient’s overall condition worsened. In addition to increasing pain, she lost appetite and weight. The histopathological results of the pancreas and liver examinations revealed a metastasized well-differentiated PanNET with expression of synaptophysin and chromogranin A (CgA). The outer membrane surface of the tumor cells expressed somatostatin receptor 2 (SSR2), and the active proliferative rate of Ki67 (a pathological grading marker) was 20 to 25% (Fig. 3). The results of additional bone marrow biopsies were unremarkable.

Extensive further diagnostic tests were done: laboratory testing and imaging with computed tomography (CT), ^68^Ga-DOTA-tyrosine octreotide (^68^Ga-DOTATOC) positron emission tomography (PET)-CT, thoracic X-ray, electrocardiography and echocardiography. Her blood work and urine analysis results are shown in Table 1.

**Table 1:** Laboratory values

Test	Value	Normal range
Glutamic oxaloacetic transaminase	277U/L	<35U/L
Glutamic pyruvic transaminase	342U/L	<35U/L
Alkaline phosphatase	233U/L	55 to 105U/L
γ-Glutamyl transferase	339U/L	<40U/L
Total bilirubin	0.7mg/dl	<1mg/dl
Conjugated bilirubin	0.26mg/dl	<0.3mg/dl
Amylase	81U/L	8 to 53U/L
Lipase	143U/L	<51U/L
Urea	2.7mg/dl	<6mg/dl
CgA	293.1U/ml	<25U/ml
NSE	26.5μg/L	<17μg/L
β-hCG	<1IU/ml	<5U/ml
AFP	<1U/ml	<8U/ml
Urine analysis	Unremarkable	

AFP, α-fetoprotein; β-hCG, Human chorionic gonadotropin, β-subunit; CgA, Chromogranin A; NSE, Neuron-specific enolase

In proximity to the previously described lesions, a widely spreading tumor with extensive surrounding of her superior mesenteric artery and her common hepatic artery was detected on imaging studies. Compression of her splenic vein and her superior mesenteric vein, as well as obstruction of her portal vein with subsequent intestine vein insufficiency and cholestasis, was also diagnosed. These anatomic findings prompted us to refrain from a (primary) curative surgical approach because of possible life-threatening circumstances.

On the basis of these diagnostic test results, an interdisciplinary decision was made to downsize the tumor with an intra-arterial 4-GBq Lu-177/4-GBq yttrium (Y)-90-DOTATOC therapy in combination with an oral radiosensitizing chemotherapy with temozolomide (5 days) and thalidomide (4 days) as an individual approach 2 weeks later. Because of an expected aplasia and the main concern of weakening her overall condition, as well as to avoid endangering the planned extended surgical procedures, cisplatin and etoposide were not used.

Despite these interventions, her condition continued to worsen. Acute pancreatitis and biliary complications developed. Analgesic treatment failed to relieve her pain. As a result, surgery was indicated, despite the increased risk for potentially fatal complications.

**Fig. 4:**
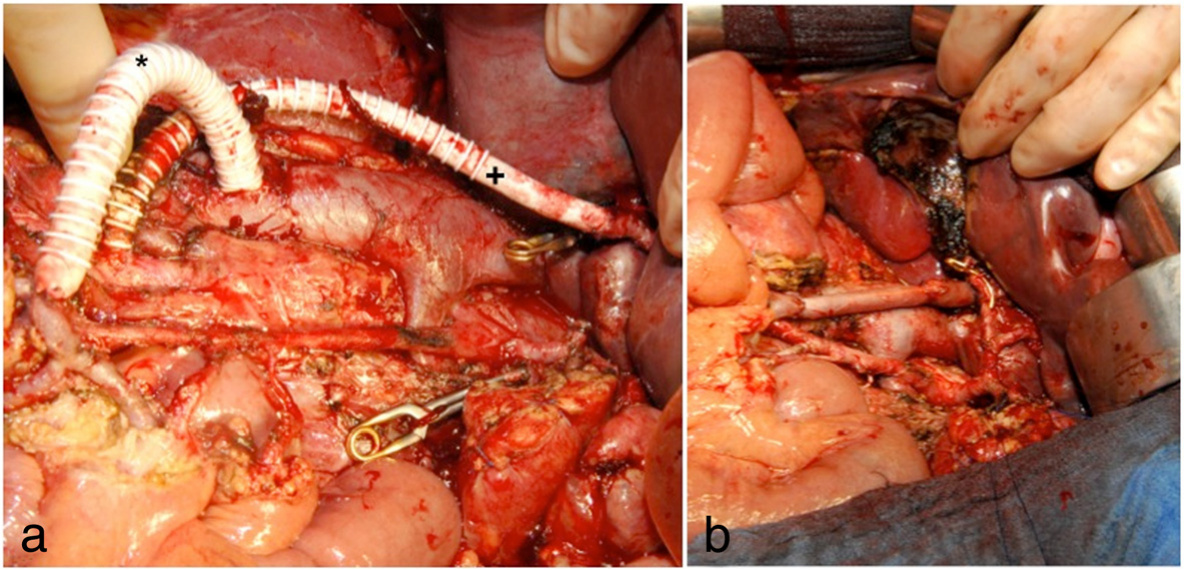
Situs. **a** Intraoperative photograph of temporal placement of a mesenteric-caval GORE-TEX (*) shunt (GORE Medical, Flagstaff, AZ, USA) and a temporal arterial aortohepatic shunt (*+*). **b** Intraoperative photograph taken after extended resection

Surgery with tumor resection took place in the same month. The following steps were performed: (1) extended pancreatic head resection, duodenectomy and cholecystectomy; (2) placement of a temporary GORE-TEX mesenteric-caval shunt and a temporary arterial aortohepatic shunt; (3) distal gastric resection with reconstruction with gastrojejunostomy (end to side), pancreaticojejunostomy and hepaticojejunostomy; (4) right hemicolectomy with terminal ileostomy and Hartmann procedure; (5) extended soft tissue resection, including the whole mesenteric root; (6) resection of the portal vein with vessel interpolation by the femoral superficial vein (from left adductor channel); (7) reconstruction of the hepatic artery with end-to-end anastomosis on the celiac trunk; and (8) atypical segmental liver resection of one metastasis (segment 2/3) (Fig. 4).

The patient recovered without major complications and could finally be dismissed with home parenteral nutrition 4 weeks later.

The first DOTATOC-PET scan obtained postoperatively showed two remaining small, right-sided liver metastases in segments 6 and 8.

Six weeks later, a right hemihepatectomy without touching the primarily made biliodigestive anastomosis was performed as planned, followed by another DOTATOC-PET scan 1 month later. We detected that the patient was having a good response to therapy without signs of residual tumor. However, splenomegaly with high DOTATOC uptake was diagnosed. Because of the importance of the nucleotide therapy with aspired further consolidating circles and the vanished option for intra-arterial application owing to the resective surgery, splenectomy was the only opportunity to guarantee successful intravenous DOTATOC therapy. Therefore, a splenectomy was performed. In the same procedure, the ileostomy was taken down.

The next intravenous Lu-177/DOTATOC therapy was done. In the same month (6 months after the histopathological specimen results), an oral tyrosine kinase inhibitor medication with sunitinib was initiated. A follow-up examination 2 months later with DOTATOC-PET and MRI scans was unremarkable. The patient recovered in a rehabilitation unit without being dependent on parenteral nutrition any longer.

**Fig. 5:**
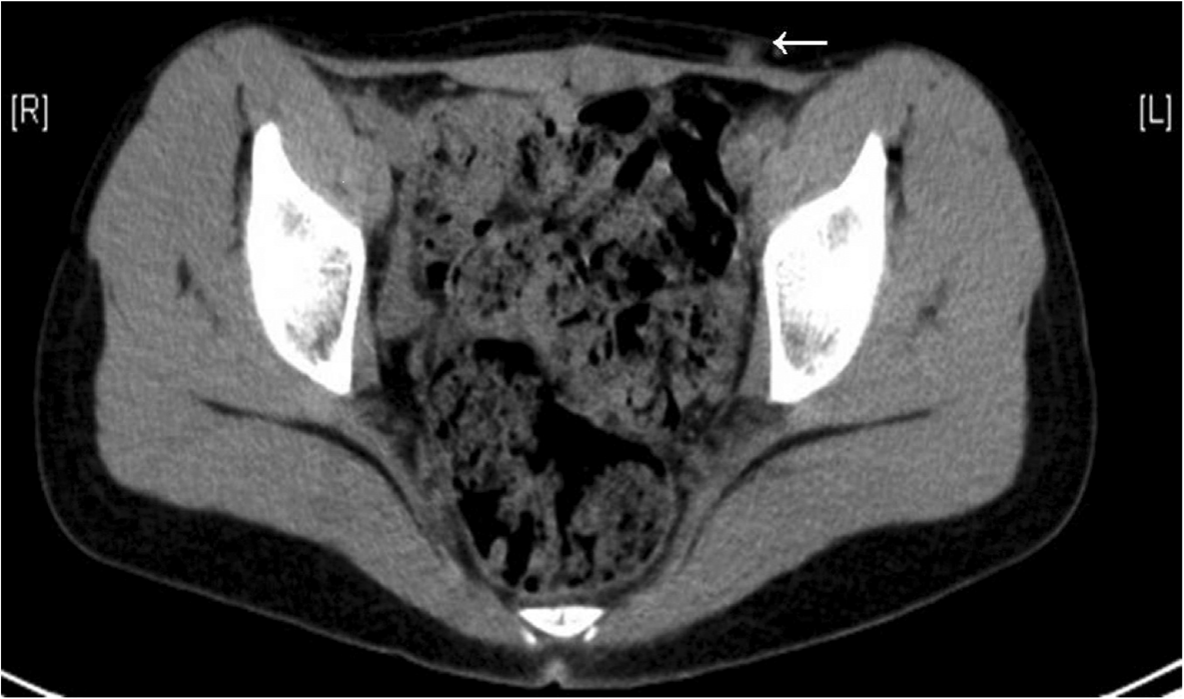
Axial computed tomographic scan without contrast agent displaying left inguinal soft tissue. Suspicious hyperdense, irregularly shaped lesion (*arrow*) with a diameter of approximately 8mm in the left inguinal soft tissue

Unfortunately, the next follow up DOTATOC-PET scan taken 1 year later showed increased uptake in her para-aortal lymph nodes and left inguinal soft tissue, so metastases of the primary tumor were expected (Fig. 5). Surgical extirpation of the suspicious left-sided inguinal lesion confirmed this hypothesis. The same endocrine malignant tumor cells could be seen (Ki-67: 30%; SSR2/DOTATOC-positive). Consequently, a para-aortic lymphadenectomy was performed.

Afterward, the patient additionally received intravenous Y-90-DOTATOC therapy. Despite those interventions, the next DOTATOC-PET scan still showed suspicious para-aortic lymph nodes. Repeat resection of the para-aortic lymph nodes was performed, followed by intravenous Ac-225-DOTATOC therapy.

Owing to the stable oncologic situation and regarding the cumulative dose of radioactivity, with its increasing risk of chronic nephrotoxicity and bone marrow toxicity, no further nuclear medical therapy was planned as of this writing. Meanwhile, somatostatin (SST) analogue therapy with Somatuline Autogel® 60mg (IPSEN, Signes, France), a long-lasting lanreotide option (injected subcutaneously once per month), as a non-radioactive blockade of tumor receptors was started to achieve remission.

The follow-up screening with DOTATOC-PET/CT 1.5 years after her first diagnosis indicated nonspecific nuclide uptake in her right breast and in one left-sided inguinal lymph node, as well as new high nuclide uptake in the right temporobasal part of her brain. Subsequent MRI of her brain confirmed a noticeable lesion raising strong suspicion of cranial metastases. Those lesions were irradiated with the patient in stereotactic positioning, and a good response was observed on the next MRI. In another surgical intervention, the left-sided inguinal lymph node was removed, which was pathologically confirmed as a metastasis of the primary tumor.

The patient reports that she is unaffected and repeat imaging is scheduled at short intervals to ensure regular follow-up.

**Fig. 6:**
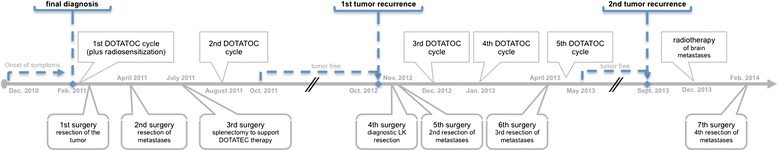
Timeline of the patient’s history. *DOTATOC* ^68^Ga-DOTA-tyrosine octreotide, *LK* lymph nodes

The case presentation is summarized in Fig. 6.

## Discussion

PanNETs are rare pancreatic neoplasms compared with their more common exocrine counterparts. Arising from the pancreatic islets of Langerhans, they are also called islet cell tumors*.* They occur in 1 to 2 of 100,000 cases per year, accounting for less than 2% of all digestive malignant tumors and less than 1% of endocrine tumors. PanNETs represent approximately 3% of primary pancreatic neoplasms. Although they may manifest at any age, they most often occur in the fourth to sixth decades of life [1, 2].

Classically, PanNETs are divided into two groups: functioning pancreatic neuroendocrine tumors (F-PanNETs) and non-functioning pancreatic neuroendocrine tumors (NF-PanNETs). F-PanNETs are hormonally active and may lead to earlier diagnosis because of their hormone-related symptoms. Common types are insulinoma and gastrinoma, and rare types are VIPoma (vasoactive intestinal peptide), glucagonoma or somatostatinoma. For example, insulinomas often present with hypoglycemic symptoms and low blood glucose levels and are reversible after glucose intake; gastrinomas present with diarrhea, hypergastrinemia, gastric acid hypersecretion and peptic ulcer diathesis, known as Zollinger–Ellison syndrome [2].

Although NF-PanNETs secrete a number of substances such as chromogranin, synaptophysin, neuron-specific enolase, pancreatic polypeptide and ghrelin, in contrast to F-PanNETs they do not present clinically with hormone-related symptoms. As a result, they often present later in the course of the disease with symptoms such as abdominal pain (35 to 75%), weight loss (20 to 35%) and anorexia and nausea (45%). Less frequent signs include obstructive jaundice (17 to 50%), intra-abdominal hemorrhage (4 to 20%) or a palpable mass (7 to 40%). Symptoms may also be attributable to metastatic disease, which occurs in, for example, the liver or bones (between 32 and 73% of cases are metastatic at diagnosis). NF-PanNETs may occur in association with the multiple endocrine neoplasia type 1 syndrome, von Hippel–Lindau syndrome, neurofibromatosis type 1 and tuberous sclerosis [2–4]. Genetic analyses were not performed in our patient. Nevertheless, considering the association of PanNETs with familial syndromes, this is an important issue, especially in young patients with PanNETs.

There are different possibilities for a diagnostic strategy: abdominal ultrasound, endoscopic ultrasound, abdominal CT, abdominal MRI, SST receptor scintigraphy, ^68^Ga-DOTATOC-PET/CT with a higher spatial resolution than scintigraphy, fine-needle aspiration cytology and/or biopsy, and laboratory tests with CgA [5, 6].

The 2010 World Health Organization classification system distinguishes between poorly differentiated neuroendocrine carcinomas (NECs) versus well-differentiated neuroendocrine tumors (NETs). All NECs are graded G3 with a Ki67 index greater than 20%. NETs are divided into G2 grade with a Ki67 index of 3 to 20% and G1 grade with a Ki67 index of 2% or less. Most pancreatic non-functioning (NF) neuroendocrine neoplasms are well differentiated (that is, NETs); NF-NECs are uncommon [3].

In the present case, our patient has been diagnosed with a well-differentiated NF-NEC, despite a Ki67 index of 20 to 25%, with a TNM classification of ypT3, ypN1(8/31), ypM1(liver) according to European Neuroendocrine Tumor Society consensus guidelines. Poorly differentiated pancreatic neuroendocrine neoplasms are characterized by their aggressive tumor biology, absence of SST receptors and poor prognosis.

Clinical management involves a multidisciplinary approach, but surgery remains the only curative therapy for early-stage disease. It represents the treatment of choice in cases of localized PanNETs, including typical and atypical resections. In particular, radical resection of pancreatic tumors is associated with favorable long-term outcomes in children and adolescents [7]. Palliative surgery of locally advanced PanNETs is justified in selected patients. For patients with metastatic NF tumors, surgery of the primary tumor is recommended only for G1 and G2.

Further treatment options are SST analogues (for example, octreotide or lanreotide) for subgroups of patients with slowly progressive, low proliferative PanNETs; systemic chemotherapy (such as streptozotocin, 5-fluorouracil or doxorubicin) in all PanNETs and NECs; peptide radionuclide receptor therapy (PRRT; SST analogues labeled with β-emitting radionuclides such as Y-90-DOTATOC) for patients with NETs with the presence of SST receptors; hepatic artery embolization or chemoembolization in patients with liver metastases who are not candidates for surgical resection; radiofrequency ablation in patients with unresectable metastases more than 5 to 7cm in diameter; radiotherapy using high-energy isotope treatment for patients with inoperable disease; immunotherapy (for example, interferon, dendritic cell immunotherapy), which may be used to offer hormone-related symptom control to patients in whom SST analogue therapy has failed; and molecular targeted therapy (including anti-epidermal growth factor receptor or anti-vascular endothelial growth factor receptor therapy such as sunitinib) for individual attempts and future options [1, 3, 8].

In our patient, a primary curative surgical approach could not be used, owing to the anatomic findings of the spreading tumor. As the initial PRRT (with Y-90-DOTATOC) combined with chemotherapy (temozolomide and thalidomide) failed [9], surgery convincingly emerged as the patient’s last chance of potentially surviving. Successful treatments to that point of the patient’s medical history involved different therapy options mainly combined repeated cycles of intravenous DOTATOC therapy with different surgical attempts, including resection of metastases and radiotherapy of the brain metastases. As shown, the patient’s best chance of surviving is an individual approach combining all therapeutic options, and, in our patient, radical surgery was the most important part.

The median overall survival for patients with NF-PanNETs is reportedly approximately 38 months. Patients with distant metastases have a median survival of 23 months, as compared with 70 to 124 months for those with localized disease [10–12]. Our patient is living and has a good quality of life approximately 42 months since the time of her first diagnosis.

## Conclusions

PanNETs are rare tumors that often invade adjacent organs and anatomic structures, causing symptoms. Because of its rarity, randomized controlled studies have not been done and current treatment recommendations are based primarily on case series and individual treatment approaches. So far, total resection of the tumor is the treatment of choice and seems to be associated with the best prognosis, particularly when accompanied by further different therapies as mentioned above. Unresectable or partially resectable tumors and recurrent tumors can be treated with local and/or systemic radiation (PRRT) and/or adjuvant chemotherapy to downsize the tumor. Even then, surgery seems to be an important intervention option, which gives the patient relief from symptoms.

## Consent

Written informed consent was obtained from the patient’s legal guardian (and herself) for publication of this case report and accompanying images. A copy of the written consent is available for review by the Editor-in-Chief of this journal.

## Abbreviations

CT: Computed tomography
DOTATOC: DOTA-tyrosine octreotide
F-PanNET: Functioning pancreatic neuroendocrine tumor
MRI: Magnetic resonance imaging
NEC: Neuroendocrine carcinoma
NET: Neuroendocrine tumor
NF: Non-functioning
NF-PanNET: Non-functioning pancreatic neuroendocrine tumor
PanNET: Pancreatic neuroendocrine tumor
PET: Positron emission tomography
PRRT: Peptide receptor radionuclide therapy
SSR2: Somatostatin receptor 2
SST: Somatostatin
Y: Yttrium
